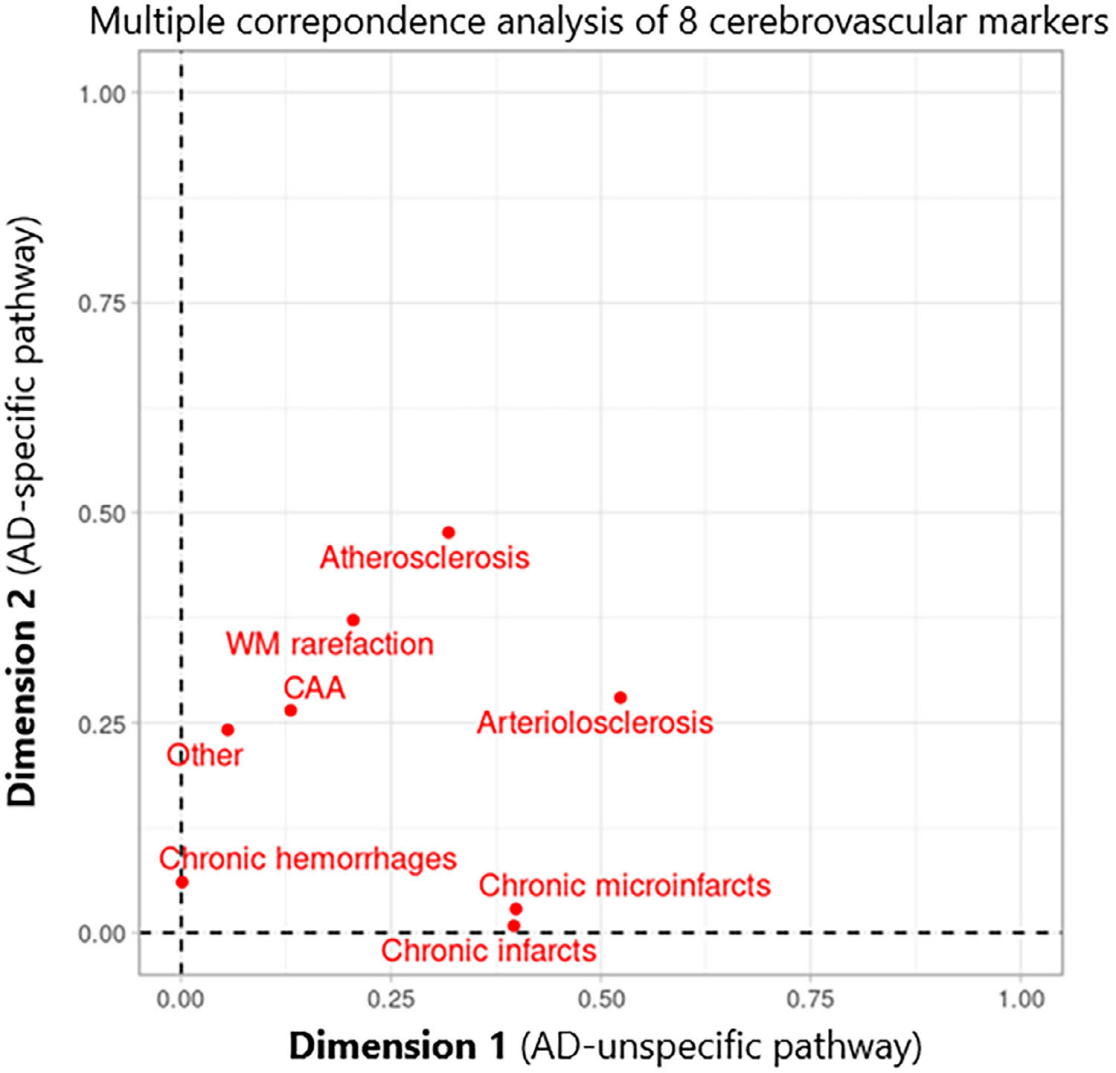# Distinct cerebrovascular pathways underlying Alzheimer’s disease‐related neurodegeneration

**DOI:** 10.1002/alz.090698

**Published:** 2025-01-03

**Authors:** Rosaleena Mohanty, Anna Marseglia, Eric Westman

**Affiliations:** ^1^ Division of Clinical Geriatrics, Center for Alzheimer Research, Department of Neurobiology, Care Sciences and Society, Karolinska Institutet, Stockholm Sweden

## Abstract

**Background:**

Cerebrovascular pathology is an emerging key contributor to neurodegeneration in both non‐pathological and pathological aging. Cerebrovascular pathology manifests as different markers in the brain (gross‐ vs micro‐infarcts, chronic vs acute infarcts, etc.). The relative association or dissociation of these markers with Alzheimer’s disease (AD) pathologies, copathologies and neurodegeneration remains poorly understood. Thus, we investigated how distinct cerebrovascular markers relate to AD pathology, copathologies, and neurodegeneration.

**Method:**

We included 77 individuals with postmortem neuropathologic examination and *in vivo* MRI (antemortem‐to‐postmortem interval = 5±3 years) from the Alzheimer’s Disease Neuroimaging Initiative. We investigated eight markers of cerebrovascular burden at postmortem including atherosclerosis of the circle of Willis, cerebral amyloid angiopathy (CAA), gross chronic infarcts, gross chronic hemorrhages, chronic microinfarcts, arteriolosclerosis in subcortical white or gray matter, white matter (WM) rarefaction and other vascular changes (acute infarcts, vascular malformation, etc.). Using multiple correspondence analysis, we identified a lower dimensional representation and assessed contributions of these eight markers. Further, we correlated the identified dimensions with other neuropathologic changes (Aβ, tau, neuritic plaques, Lewy body, TDP‐43, hippocampal sclerosis, and atrophy) and antemortem MRI measures (total gray matter volume and white matter hypointensity volume).

**Result:**

Individuals were 83±7 years‐old at death and included 26% women, 56% APOE‐ε4 carriers and 75% individuals with confirmed AD neuropathologic change. Multiple correspondence analysis on the eight cerebrovascular markers resulted in two dimensions (**Fig. 1**). Dimension 1 was characterized by presence of gross chronic infarcts, chronic microinfarcts, mild WM rarefaction, severe arteriosclerosis, and severe atherosclerosis. Dimension 1 was significantly associated with higher postmortem cortical atrophy (*r* = 0.2), hippocampal atrophy (*r* = 0.4), hippocampal sclerosis (*r* = 0.3) and antemortem WM hypointensity volume (*r* = 0.3). Dimension 2 was characterized by moderate atherosclerosis, moderate arteriosclerosis, moderate WM rarefaction, severe CAA, presence of chronic hemorrhages and other vascular changes (acute gross‐/micro‐infarcts, microhemorrhage, vasculitis, blood vessel mineralization). Dimension 2 was significantly associated with higher postmortem Aβ (*r* = ‐0.4), tau (*r* = ‐0.3), neuritic plaques (*r* = ‐0.3) and hence, AD neuropathologic change (*r* = ‐0.3).

**Conclusion:**

We identified two data‐driven cerebrovascular pathways, one AD‐unspecific (dimension 1) and one AD‐specific (dimension 2). Cerebrovascular burden may differentially manifest in individuals depending on the underlying cause of neurodegeneration.